# Risk Factors and Survival of Patients With Liver Metastases at Initial Metastatic Breast Cancer Diagnosis in Han Population

**DOI:** 10.3389/fonc.2021.670723

**Published:** 2021-05-11

**Authors:** Shaoyan Lin, Hongnan Mo, Yiqun Li, Xiuwen Guan, Yimeng Chen, Zijing Wang, Peng Yuan, Jiayu Wang, Yang Luo, Ying Fan, Ruigang Cai, Qiao Li, Shanshan Chen, Pin Zhang, Qing Li, Fei Ma, Binghe Xu

**Affiliations:** ^1^ Department of Medical Oncology, National Cancer Center/National Clinical Research Center for Cancer/Cancer Hospital, Chinese Academy of Medical Sciences & Peking Union Medical College, Beijing, China; ^2^ Department of VIP Medical Services, National Cancer Center/National Clinical Research Center for Cancer/Cancer Hospital, Chinese Academy of Medical Sciences & Peking Union Medical College, Beijing, China

**Keywords:** metastatic breast cancer, incidence, risk factors, liver metastases, survival

## Abstract

The risk factors for morbidity and mortality in patients with breast cancer liver metastases (BCLM) upon initial metastatic breast cancer (MBC) diagnosis have not been adequately identified in Han population. Data of 3,161 female patients who were initially diagnosed with MBC from December 1991 to September 2019 and treated in the China National Cancer Center were extracted and a total of 2,263 MBC patients were included in our study, among which 550 patients had liver metastases. Multivariable logistic regression was performed to identify risk factors for the presence of liver metastases at initial MBC diagnosis. Univariable and multivariable Cox proportional hazards regression analyses were conducted to determine prognostic factors for the survival of BCLM patients. Patients with hormone receptor (HR)-negative, human epidermal growth factor receptor 2 (HER2)-positive (35.0% of the entire population) subtype had the highest incidence of liver metastases. *De novo* stage IV breast cancer, HR−/HER2+ and HR+/HER2+ subtypes were associated with higher odds of liver metastases and patients with lung metastases had lower risk of liver metastases at initial MBC diagnosis. The median overall survival of BCLM patients was 31.4 months and BCLM patients with HR+/HER2− subtype had the longest survival of 38.2 months. Older age, worse performance status, later stage of initial breast cancer, triple-negative subtype and lung metastases were significantly associated with a poorer prognosis in BCLM patients. Our study offers insights into the incidence and prognosis of BCLM patients at initial MBC diagnosis in Han population.

## Introduction

Breast cancer is the most common cancer and the leading cause of cancer death in female worldwide (1). It is reported that ~6% of breast cancer patients present with *de novo* metastatic disease and ~30% of patients with early-stage breast cancer will eventually recur (2). With a median survival time of ~3 years, metastatic breast cancer (MBC) remains incurable (3).

Liver metastasis is one of the most frequent distant metastases of breast cancer (4), with an incidence of ~30% (5, 6) in MBC patients. Earlier studies have reported that the outcome of patients with breast cancer liver metastases (BCLM) is usually poor, and the median survival is 12–20 months (7–9). The tumor subtypes are demonstrated to be associated with the prognosis of BCLM patients and triple-negative breast cancer (TNBC) confers the shortest survival when compared with other subtypes (10). Additionally, growing evidence implies that the distinct molecular subtypes show preferential sites of recurrence (4, 11). Breast cancer patients with human epidermal growth factor receptor 2 (HER2) overexpression are more likely to develop liver metastases compared with HER2-negative patients (12). However, the risk factors and survival in patients with BCLM upon initial MBC diagnosis in Han population remain poorly identified.

In this study, we aimed to investigate the risk factors for morbidity and mortality of liver metastases in newly diagnosed MBC patients in Han population. We also characterized clinicopathological features and overall survival (OS) of BCLM patients according to breast cancer subtype.

## Materials and Methods

### Patients

We retrospectively extracted data of 3,161 female patients who were initially diagnosed with MBC from December 1991 to September 2019 and treated in the China National Cancer Center. Patients with unknown hormone receptor (HR) or HER2 status (n = 579), unknown metastatic sites (n = 65) and follow-up less than 1 month from initial MBC diagnosis (n = 254) were excluded, leaving 2,263 patients in the final cohort eligible for incidence analysis. Among these, 550 patients had liver metastases when first diagnosed with MBC. All included participants were followed until June 30, 2019 or date of deaths by telephone contacts or outpatient visits.

### Study Variables

We collected the following clinical data of included patients from medical records in hospital information system: age at MBC diagnosis, Eastern Cooperative Oncology Group (ECOG) score, initial stage of breast cancer, tumor subtype, site of metastases and survival month. HR status was measured by routine immunohistochemistry (IHC) and cancers with 1–100% estrogen receptor IHC staining or 1–100% progesterone receptor IHC staining were considered HR-positive. HER2 IHC3+ or amplified fluorescent *in situ* hybridization (FISH) were reported HER2-poxitive. Patients were divided into four different subtypes: HR+/HER2−, HR−/HER2+, HR+/HER2+ and triple-negative (HR−/HER2−). Breast cancer staging was according to the 8th American Joint Committee on Cancer (AJCC) TNM staging system.

### Statistical Analysis

Categorical data was described using numbers and percentages and the chi-square test was performed to compare category variables among different breast cancer subtypes in BCLM patients. Incidence of liver metastases upon initially MBC diagnosis was calculated among the entire cohort stratified by breast cancer subtype. Multivariable logistic regression was used to determine whether age, ECOG, initial stage of breast cancer, tumor subtype, and site of extrahematic metastases were associated with the presence of liver metastases at first MBC diagnosis. Odds ratios (ORs) with 95% confidence intervals (CIs) were calculated. OS was defined as the time from the initial MBC diagnosis to death. We utilized Kaplan–Meier analysis and log-rank test to estimate the cumulative OS within subsets of breast cancer subtypes and compare the differences. Univariable and multivariable Cox proportional hazards regression analyses were conducted to identify the independent prognostic factors significantly influencing the OS of BCLM patients. Statistical analyses were performed independently by Shaoyan Lin, MD, using SPSS statistical software version 23. A two-sided *P* value <0.05 was considered as statistically significant.

## Results

### Patient Characteristics

Of the 2,263 included patients, 24.3% (550) developed liver metastases when first diagnosed with MBC. Of patients with BCLM, 20.4% (112) presented with *de novo* stage IV disease. **Table 1** summarized the clinicopathological features of BCLM patients according to breast cancer subtype. HR+/HER2−, HR−/HER2+, HR+/HER2+ and triple-negative subtypes comprised 46.0% (253), 19.1% (105), 22.4% (123) and 12.5% (69) of BCLM patients, respectively. Compared with other groups, HR+/HER2− patients with BCLM were less likely to present with *de novo* MBC (*P* = 0.000). Besides, HR+ (HR+/HER2− and HR+/HER2+) patients with BCLM had a higher rate of bone metastases than HR− (HR−/HER2+ and triple-negative) patients (*P* = 0.020).

**Table 1:** Clinicopathological features of patients with liver metastases at initial metastatic breast cancer diagnosis stratified by breast cancer subtype.

Characteristic	HR+/HER2−, N (%)	HR−/HER2+, N (%)	HR+/HER2+, N (%)	Triple-negative, N (%)	*P* value
All patients	253 (46.0)	105 (19.1)	123 (22.4)	69 (12.5)	
Age					0.887
<50	125 (49.4)	51 (48.6)	65 (52.8)	33 (47.8)	
≥50	128 (50.6)	54 (51.4)	58 (47.2)	36 (52.2)	
ECOG					0.444
0	66 (26.1)	25 (23.8)	27 (22.0)	17 (24.6)	
1	178 (70.4)	75 (71.4)	90 (73.2)	45 (65.2)	
2	9 (3.6)	5 (4.8)	6 (4.9)	7 (10.1)	
T-stage					0.094
T1	65 (25.7)	22 (21.0)	32 (26.0)	13 (18.8)	
T2	119 (47.0)	41 (39.0)	52 (42.3)	27 (39.1)	
T3	15 (5.9)	15 (14.3)	7 (5.7)	11 (15.9)	
T4	15 (5.9)	10 (9.5)	6 (4.9)	4 (5.8)	
Unknown	39 (15.4)	17 (16.2)	26 (21.1)	14 (20.3)	
N-stage					0.063
N0	64 (25.3)	26 (24.8)	21 (17.1)	18 (26.1)	
N1	67 (26.5)	31 (29.5)	23 (18.7)	11 (15.9)	
N2	52 (20.6)	19 (18.1)	35 (28.5)	14 (20.3)	
N3	45 (17.8)	23 (21.9)	29 (23.6)	13 (18.8)	
Unknown	25 (9.9)	6 (5.7)	15 (12.2)	13 (18.8)	
M-stage					0.000
M0	229 (90.5)	77 (73.3)	82 (66.7)	50 (72.5)	
M1	24 (9.5)	28 (26.7)	41 (33.3)	19 (27.5)	
Lung metastases					0.733
No	175 (69.2)	74 (70.5)	81 (65.9)	44 (63.8)	
Yes	78 (30.8)	31 (29.5)	42 (34.1)	25 (36.2)	
Brain metastases					0.353
No	246 (97.2)	98 (93.3)	118 (95.9)	67 (97.1)	
Yes	7 (2.8)	7 (6.7)	5 (4.1)	2 (2.9)	
Bone metastases					0.020
No	148 (58.5)	76 (72.4)	75 (61.0)	51 (73.9)	
Yes	105 (41.5)	29 (27.6)	48 (39.0)	18 (26.1)	

HR, hormone receptor; HER2, human epidermal growth factor receptor 2; ECOG, Eastern Cooperative Oncology Group.

### Incidence

**Table 2** listed the result of incidence of liver metastases according to breast cancer subtype among the entire cohort. Of the 2,263 patients presenting with MBC, 52.1, 13.3, 16.1 and 18.5% had HR+/HER2−, HR−/HER2+, HR+/HER2+ and triple-negative subtypes, respectively. Patients with HR-/HER2+ (35.0%) and HR+/HER2+ (33.7%) subtypes shared the highest incidence proportions of liver metastases, while triple-negative (16.5%) the lowest.

**Table 2:** Incidence of patients with liver metastases at initial metastatic breast cancer diagnosis according to breast cancer subtype.

	All metastatic patients, N (%)	With liver metastases	Incidence of liver metastases, %
HR+/HER2−	1180 (52.1)	253	21.4
HR−/HER2+	300 (13.3)	105	35.0
HR+/HER2+	365 (16.1)	123	33.7
Triple-negative	418 (18.5)	69	16.5
All subtypes	2263 (100.0)	550	24.3

HR, hormone receptor; HER2, human epidermal growth factor receptor 2.

The multivariate logistic regression for the presence of liver metastases among the entire MBC population was displayed in **Table 3**. Patients with *de novo* stage IV breast cancer were more likely to present with liver metastases than recurrent MBC patients (M1 *vs.* M0, OR = 1.44, 95% CI = 1.08–1.93, *P* = 0.013). HR−/HER2+ (*vs.* HR+/HER2−, OR = 1.92, 95% CI = 1.44–2.55, *P* = 0.000) and HR+/HER2+ (*vs.* HR+/HER2−, OR = 1.80, 95% CI = 1.38–2.34, *P* = 0.000) subtypes were associated with remarkably higher odds of liver metastases, while triple-negative subtype (*vs.* HR+/HER2−, OR = 0.71, 95% CI = 0.53–0.96, *P* = 0.024) was associated with significantly lower odds of liver metastases at first MBC diagnosis. Patients with lung metastases (*vs.* without lung metastases, OR = 0.78, 95% CI = 0.63–0.96, *P* = 0.022) had lower risk of liver metastases at diagnosis. Age, ECOG, T-stage, N-stage, brain and bone metastases showed no correlations with the presence of liver metastases at diagnosis.

**Table 3:** Multivariate logistic regression for the presence of liver metastases at initial metastatic breast cancer diagnosis.

Characteristic	OR (95% CI)	*P* value
Age		
<50	Reference	
≥50	1.04 (0.85, 1.26)	0.729
ECOG		
0	Reference	
1	1.00 (0.79, 1.26)	0.979
2	1.52 (0.91, 2.54)	0.110
T-stage		
T1	Reference	
T2	1.03 (0.80, 1.32)	0.834
T3	1.21 (0.80, 1.83)	0.359
T4	1.09 (0.66, 1.78)	0.738
Unknown	0.71 (0.51, 1.00)	0.047
N-stage		
N0	Reference	
N1	1.09 (0.82, 1.44)	0.567
N2	1.35 (0.99, 1.83)	0.055
N3	0.98 (0.71, 1.35)	0.896
Unknown	1.34 (0.88, 2.04)	0.168
M-stage		
M0	Reference	
M1	1.44 (1.08, 1.93)	0.013
Subtype		
HR+/HER2−	Reference	
HR−/HER2+	1.92 (1.44, 2.55)	0.000
HR+/HER2+	1.80 (1.38, 2.34)	0.000
Triple-negative	0.71 (0.53, 0.96)	0.024
Lung metastases		
No	Reference	
Yes	0.78 (0.63, 0.96)	0.022
Brain metastases		
No	Reference	
Yes	0.75 (0.45, 1.26)	0.276
Bone metastases		
No	Reference	
Yes	0.85 (0.69, 1.05)	0.142

OR, odds ratio; CI, confidence interval; ECOG, Eastern Cooperative Oncology Group; HR, hormone receptor; HER2, human epidermal growth factor receptor 2.

### Survival

**Figure 1:**
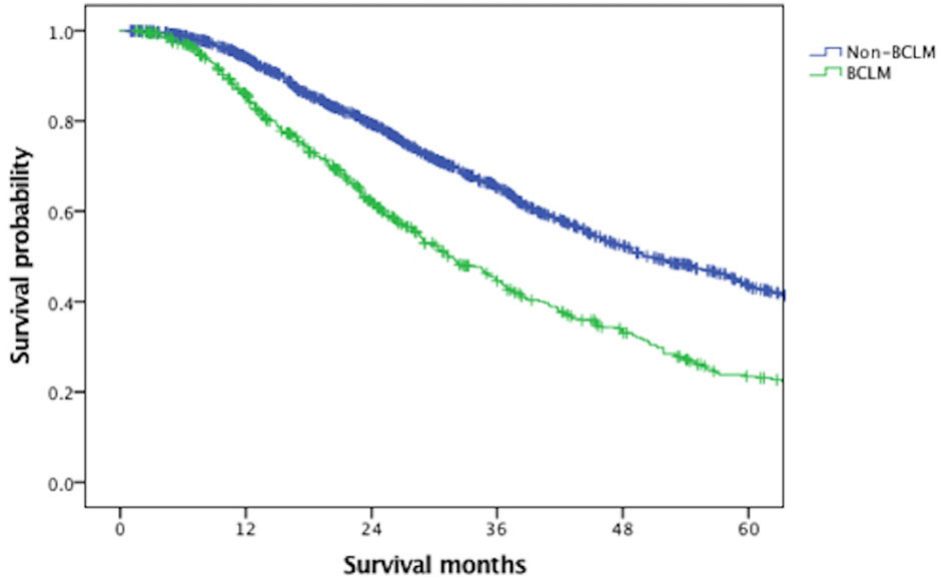
Overall survival of BCLM and non-BCLM patients among the entire population. BCLM, breast cancer liver metastases.

**Figure 2:**
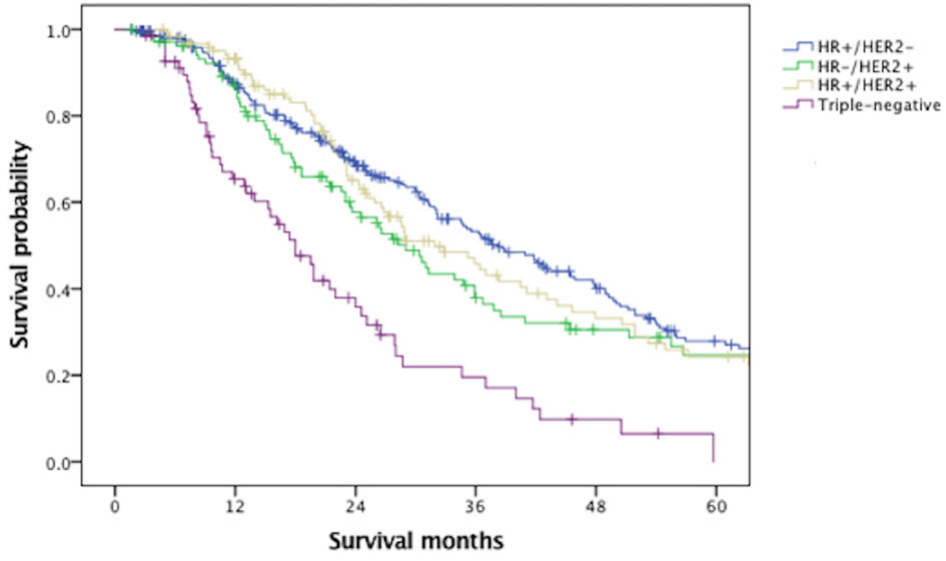
Overall survival of patients with liver metastases stratified by breast cancer subtype. HR, hormone receptor; HER2, human epidermal growth factor receptor 2.

With a median follow-up of 61.6 months, the median OS of the overall MBC population was 45.4 months. **Figure 1** indicated that BCLM patients (median OS, 31.4 months) had significantly poorer prognosis than non-BCLM patients (median OS, 50.0 months, *P* = 0.000) at first MBC diagnosis. The OS of BCLM patients stratified by breast cancer subtype was showed in **Figure 2**. BCLM patients with HR+/HER2− subtype had the longest survival (38.2 months) while triple-negative the shortest (18.0 months, *P* = 0.000). The median OS of BCLM patients with HR-/HER2+ (*vs.* HR+/HER2−, *P* = 0.282) and HR+/HER2+ (*vs.* HR+/HER2*-*, *P* = 0.518) subtypes were 29.0 and 31.9 months, respectively.

Univariable and multivariable Cox proportional hazards regression analyses were performed to assess the prognostic factors of BCLM patients (**Table 4**). The variables of age, ECOG, T-stage, N-stage, subtype, lung and bone metastases with *P <*0.05 in univariable analysis were incorporated into the multivariable regression analysis. Age ≥50 years (*vs.* <50 years, HR = 1.27, 95% CI = 1.02–1.58, *P* = 0.031), ECOG 1 (*vs.* ECOG 0, HR = 1.37, 95% CI = 1.03–1.83, *P* = 0.032), ECOG 2 (*vs.* ECOG 0, HR = 1.93, 95% CI = 1.17–3.18, *P* = 0.010), T4 (*vs.* T1, HR = 1.62, 95% CI = 1.02–2.58, *P* = 0.043), N2 (*vs.* N0, HR = 1.75, 95% CI = 1.23–2.48, *P* = 0.002), N3 (*vs.* N0, HR = 1.50, 95% CI = 1.05–2.14, *P* = 0.025), triple-negative subtype (*vs.* HR+/HER2−, HR = 2.51, 95% CI = 1.77–3.56, *P* = 0.000) and lung metastases (*vs.* without lung metastases, HR = 1.60, 95% CI = 1.27–2.03, *P* = 0.000) were significantly associated with an increased mortality.

**Table 4:** Univariable and multivariable cox regression analyses of overall survival in BCLM patients.

Univariable analysis			Multivariable analysis		
Characteristic	Hazard ratio (95% CI)	*P* value	Characteristic	Hazard ratio (95% CI)	*P* value
Age			Age		
<50	Reference		<50	Reference	
≥50	1.25 (1.01, 1.54)	0.040	≥50	1.27 (1.02, 1.58)	0.031
ECOG			ECOG		
0	Reference		0	Reference	
1	1.45 (1.09, 1.92)	0.010	1	1.37 (1.03, 1.83)	0.032
2	2.75 (1.72, 4.39)	0.000	2	1.93 (1.17, 3.18)	0.010
T-stage			T-stage		
T1	Reference		T1	Reference	
T2	1.10 (0.84, 1.45)	0.470	T2	1.01 (0.77, 1.34)	0.928
T3	1.80 (1.20, 2.70)	0.005	T3	1.11 (0.71, 1.74)	0.658
T4	2.24 (1.46, 3.45)	0.000	T4	1.62 (1.02, 2.58)	0.043
Unknown	1.02 (0.73, 1.44)	0.892	Unknown	0.93 (0.63, 1.38)	0.719
N-stage			N-stage		
N0	Reference		N0	Reference	
N1	1.16 (0.84, 1.61)	0.363	N1	1.13 (0.82, 1.57)	0.457
N2	1.92 (1.39, 2.66)	0.000	N2	1.75 (1.23, 2.48)	0.002
N3	1.71 (1.22, 2.38)	0.002	N3	1.50 (1.05, 2.14)	0.025
Unknown	1.43 (0.96, 2.13)	0.079	Unknown	1.11 (0.69, 1.76)	0.673
M-stage					
M0	Reference				
M1	1.17 (0.90, 1.53)	0.232			
Subtype			Subtype		
HR+/HER2−	Reference		HR+/HER2−	Reference	
HR−/HER2+	1.18 (0.88, 1.57)	0.271	HR−/HER2+	1.23 (0.91, 1.66)	0.181
HR+/HER2+	1.08 (0.82, 1.43)	0.576	HR+/HER2+	1.05 (0.79, 1.39)	0.755
Triple-negative	2.73 (1.98, 3.78)	0.000	Triple-negative	2.51 (1.77, 3.56)	0.000
Lung metastases			Lung metastases		
No	Reference		No	Reference	
Yes	1.86 (1.49, 2.33)	0.000	Yes	1.60 (1.27, 2.03)	0.000
Brain metastases					
No	Reference				
Yes	1.63 (0.98, 2.69)	0.058			
Bone metastases			Bone metastases		
No	Reference		No	Reference	
Yes	1.26 (1.01, 1.56)	0.037	Yes	1.21 (0.96, 1.52)	0.107

BCLM, breast cancer liver metastases; CI, confidence interval; ECOG, Eastern Cooperative Oncology Group; HR, hormone receptor; HER2, human epidermal growth factor receptor 2.

## Discussion

In this article, we described the incidence, outcome and their associated risk factors of BCLM patients in newly diagnosed MBC patients in Han population. We identified 550 cases of liver metastases from initially diagnosed MBC patients, accounting for 24.3% of the entire cohort. Consistently, previous studies (10, 13) reported that 25.0–29.6% of the advanced breast cancer patients had the initial recurrence in liver. **Table 1** indicated that compared with other subsets, HR+/HER2− patients with BCLM were more likely to present with recurrent disease, rather than *de novo* MBC (*P* = 0.000), which were not found in the whole MBC population in earlier studies (14, 15). Besides, HR-positive patients with BCLM had a higher rate of bone metastases than HR-negative patients (*P* = 0.020), similar to previous studies (4, 16).

In accordance with other work (9, 12, 17–19), our study demonstrated that patients with HER2-positive had the highest incidence of liver metastases (**Table 2**). A retrospective study including 3,276 patients with BCLM in the USA also indicated that patients with HR−/HER2+ (46.5%) and HR+/HER2+ (37.5%) subtypes had a propensity for liver metastases compared with HER2-negative subtypes (9). Some studies tried to figure out the molecular mechanisms associated with specific metastatic sites in breast cancer. Pierobon and colleagues found that increased incidence of *PIK3CA* mutations and activation of PI3K-AKT-mTOR signaling axis, related to HER2 activation, were involved in liver metastases in breast cancer (20). Li et al. reported that HER2 enhanced the expression of CXCR4, a chemokine receptor, thus mediating the breast tumor metastasis to specific organs (21). PRL-3, a phosphatase promoting cell migration, invasion and liver metastasis, was found to positively express in HER2-positive breast cancers (22). Further studies to clarify the molecular mechanisms of HER2-mediated breast tumor metastasis to liver may provide new targeted agents for any individual patient.

The multivariate logistic regression analysis **(** **Table 3**) showed that patients with *de novo* stage IV breast cancer were more prone to present with liver metastases than recurrent MBC patients, which was also found in two prospective observational cohort studies conducted in America (23, 24). Besides, patients with lung metastases had smaller likelihood of liver metastases at diagnosis. In other words, liver metastases were less likely to happen with lung metastases simultaneously upon initial diagnosis of MBC. This difference of metastatic sites might be due to the molecular and cellular mechanisms underlying organ-specific metastases. Breast cancer expression of DAP12, a transmembrane adapter protein, was reported to promote skeletal and liver metastases, but not lung metastases (25). Early detection of biomarkers involving in tissue-specific metastases may be beneficial to clinical determination. Therefore, more mechanistic studies associated with organ metastases in breast cancer are desirable for clinical practice.

The median OS of BCLM patients was 31.4 months, remarkably poorer than non-BCLM patients, the median OS of whom was 50.0 months. The survival of BCLM patients in our data seemed longer than the reported survival of BCLM patients in former retrospective studies, ranging from 12 to 20 months (7–10). Additionally, we observed that BCLM patients with triple-negative subtype had the shortest survival (18.0 months) compared with other subtypes, consistent with previous publications (17, 26, 27). In a California cohort of 6,268 *de novo* MBC patients, the median survival of TNBC patients was much shorter (12 months) than other subtypes (28). There was no significant difference in outcome among HR+/HER2− (38.2 months), HR−/HER2+ (29.0 months) and HR+/HER2+ (31.9 months) subtypes, probably thanks to the dramatically improved anti-HER2 therapies (29).

We also determined independent risk factors for survival of BCLM patients using univariable and multivariable analyses (**Table 4**). Performance status, age, stage of initial breast cancer have been proven to be prognostic parameters in multiple studies (30–32), similar to our results. Additionally, the presence of extrahepatic metastases was demonstrated to worsen outcome in BCLM patients (33, 34). In our study, BCLM patients presenting with lung metastases simultaneously had a more unfavorable survival than those without lung metastases. Similar tendency was seen in patients developing brain metastases but the result did not reach significance, which might be due to the rare occurrence of brain metastases at initial MBC diagnosis (35).

Our study had some limitations. Firstly, reassessment of the metastatic tumor receptor status was beneficial since changes in tumor phenotype have been found between primary and recurrent breast cancer (36). Secondly, there was no record of the extension and lesion of liver metastases, which were important prognostic factors for BCLM patients (26). Finally, the retrospective setting of this study required a multicenter, large-scale and prospective research to confirm the results.

In summary, our study offers insights into the incidence and prognosis of BCLM patients at initial MBC diagnosis in Han population. It summarizes the clinicopathological features of BCLM patients stratified by breast cancer subtype and helps to identify MBC patients with high risk of liver metastases. Additionally, it provides essential information on prognostic factors of BCLM patients, which is beneficial to personalized treatment in clinical practice. Further strategies covering early screening and prognosis evaluation for BCLM patients are warranted to optimize the disease outcome.

## Data Availability Statement

The raw data supporting the conclusions of this article will be made available by the authors, without undue reservation.

## Ethics Statement

Written informed consent was not obtained from the individual(s) for the publication of any potentially identifiable images or data included in this article.

## Author Contributions

Conception and design: BX. Assembly of data: All authors. Data analysis and interpretation: SL. Manuscript writing: All authors. All authors contributed to the article and approved the submitted version.

## Funding

This work was supported by National Key R&D Program of China (2018YFC1312101) and Chinese Academic of Medical Sciences Initiative for Innovative Medicine (CAMS-12M-1-010).

## Conflict of Interest

The authors declare that the research was conducted in the absence of any commercial or financial relationships that could be construed as a potential conflict of interest.

## Abbreviations

AJCC, American Joint Committee on Cancer; BCLM, breast cancer liver metastases; CI, confidence interval; ECOG, Eastern Cooperative Oncology Group; FISH, fluorescent *in-situ* hybridization; HER2, human epidermal growth factor receptor 2; HR, hormone receptor; IHC, immunohistochemistry; MBCm metastatic breast cancer; OR, Odds ratio; OS, overall survival; TNBC, triple-negative breast cancer.
